# Short-term effects of stored homologous red blood cell transfusion on cardiorespiratory function and inflammation: an experimental study in a hypovolemia model

**DOI:** 10.1590/1414-431X20176258

**Published:** 2017-11-17

**Authors:** S. Biagini, C.S. Dale, J.M. Real, E.S. Moreira, C.R.R. Carvalho, G.P.P. Schettino, S. Wendel, L.C.P. Azevedo

**Affiliations:** 1 Instituto de Ensino e Pesquisa, Hospital Sírio-Libanês, São Paulo, SP, Brasil; 2 Laboratorio de Neuromodulação e Dor Experimental, Departamento de Anatomia, Universidade de São Paulo, São Paulo, SP, Brasil; 3 Associação TUCCA para Crianças e Adolescentes com Câncer, Departamento de Oncologia Pediátrica, Hospital Santa Marcelina, São Paulo, Brasil; 4 Centro de Investigação Translacional em Oncologia, Instituto do Câncer do Estado de São Paulo, Universidade de São Paulo, São Paulo, SP, Brasil; 5 Hospital do Servidor Público Estadual de São Paulo (IAMSPE), São Paulo, SP, Brasil; 6 Evidências - Kantar Health, São Paulo, SP, Brasil; 7 Departamento de Cardiopneumologia, Instituto do Coração, Universidade de São Paulo, São Paulo, SP, Brasil; 8 Hospital Municipal da Vila Santa Catarina, Sociedade Beneficente Israelita Albert Einstein, São Paulo, SP, Brasil; 9 Banco de Sangue, Hospital Sirio-Libanes, São Paulo, SP, Brasil; 10 Disciplina de Emergências Clínicas, Universidade de São Paulo, São Paulo, SP, Brasil

**Keywords:** Swine, Transfusion, Inflammation, Red blood cells, Hypovolemia, Hemorrhage

## Abstract

The pathophysiological mechanisms associated with the effects of red blood cell (RBC) transfusion on cardiopulmonary function and inflammation are unclear. We developed an experimental model of homologous 14-days stored RBC transfusion in hypovolemic swine to evaluate the short-term effects of transfusion on cardiopulmonary system and inflammation. Sixteen healthy male anesthetized swine (68±3.3 kg) were submitted to controlled hemorrhage (25% of blood volume). Two units of non-filtered RBC from each animal were stored under blood bank conditions for 14 days. After 30 min of hypovolemia, the control group (n=8) received an infusion of lactated Ringer's solution (three times the removed volume). The transfusion group (n=8) received two units of homologous 14-days stored RBC and lactated Ringer's solution in a volume that was three times the difference between blood removed and blood transfusion infused. Both groups were followed up for 6 h after resuscitation with collection of hemodynamic and respiratory data. Cytokines and RNA expression were measured in plasma and lung tissue. Stored RBC transfusion significantly increased mixed oxygen venous saturation and arterial oxygen content. Transfusion was not associated with alterations on pulmonary function. Pulmonary concentrations of cytokines were not different between groups. Gene expression for lung cytokines demonstrated a 2-fold increase in mRNA level for inducible nitric oxide synthase and a 0.5-fold decrease in mRNA content for IL-21 in the transfused group. Thus, stored homologous RBC transfusion in a hypovolemia model improved cardiovascular parameters but did not induce significant effects on microcirculation, pulmonary inflammation and respiratory function up to 6 h after transfusion.

## Introduction

Blood transfusion is an established treatment for several life-threatening conditions during critical illness. Apart from life-threatening scenarios, however, there is controversy on when to transfuse a hemodynamically stable critically ill patient. Possible adverse events of transfusion include induction of inflammatory response and pulmonary dysfunction (1–3). In addition, long-term preservation of red blood cells (RBC) under blood bank conditions may result in the accumulation of inflammatory mediators in the storage medium and increased levels of free hemoglobin, which can scavenge nitric oxide (NO). These changes could be related to occurrence of microvascular vasoconstriction, platelet activation, and pro-inflammatory and pro-oxidant activity described in transfused patients (4).

Some clinical studies have found an association of blood transfusion with organ failure, longer hospitalizations and increased mortality (5–9). However, other studies did not find the same association (10–12). An important limitation of these data is their observational study design with the unavoidable potential for bias and residual confounding (13–15). These biases may come from the disease severity of the patients included in trials and the presence of frequent comorbidities such as sepsis and trauma. Despite the use of statistical techniques that may adjust for these and other potential confounders, it is very difficult to establish the independent role of transfusion in adverse outcomes in these settings (15). On the other hand, clinical studies to test blood transfusion effects in healthy people would be ethically unacceptable.

Complications involving the respiratory system are described as important causes of transfusion-related morbidity (16). In newborns, transfusion induced a reduction of respiratory system compliance and an increase in pulmonary vascular resistance (17). In addition, in mechanically ventilated premature infants, blood transfusion increased the concentrations of malondialdehyde (a marker of oxidative stress) on bronchoalveolar lavage (18). A study done to evaluate the increase in oxygen consumption after RBC transfusion in septic patients also showed significant increases in respiratory system resistance when compared to albumin infusion (19). In a recent study in intensive care patients on mechanical ventilation, a RBC transfusion of one unit did not induce differences in lung function, as well as in the inflammatory and coagulation status after 2 h (20). Therefore, the results are unclear and may be caused by different clinical scenarios, presence of comorbidities, variable number of transfused units and storage time.

In this context, animal studies may partially overcome these limitations, since healthy animals can be evaluated to identify possible harmful mechanisms of transfusion. The swine model has been extensively tested in critical care since there are clear similarities between these animals and human beings mainly regarding cardiopulmonary function (15,21,22). In this study, we evaluated the effect of transfusion on an experimental model without any other clinically significant insult than RBC administration, with the purpose of assessing the independent effect of blood products. We, therefore, developed a clinically relevant experimental model of RBC transfusion and we examined the immediate impact of this strategy on hemodynamics, microcirculation, pulmonary function and systemic and pulmonary inflammation.

## Material and Methods

This study was approved by the Institutional Animal Research Ethics Committee from Hospital Sírio-Libanês and was performed according to the National Institute of Health Guidelines for the use of experimental animals.

### Instrumentation and stabilization period

Sixteen domestic Agroceres^®^ male pigs (body weight, 68±3.3 kg) were fasted overnight with free access to water and pre-medicated with an intramuscular injection of midazolam (0.3 mg/kg) and acepromazine (0.5 mg/kg). Anesthesia was induced with thionembutal (12 mg/kg) and muscular relaxation with pancuronium bromide (0.1 mg/kg). The animals were submitted to endotracheal intubation and mechanical ventilation (Evita XL, Dräger, Germany) with the following baseline ventilatory settings: tidal volume (VT): 8 mL/kg, positive end-expiratory pressure (PEEP) of 5 cmH_2_O, peak airway flow of 1 L/s, inspiratory fraction of oxygen adjusted to maintain arterial saturation between 93-95% and respiratory rate necessary to maintain PaCO_2_ between 35 and 45 mmHg. Anesthesia was maintained during the study with midazolam (0.3 mg·kg^-1^·h^-1^) and fentanyl citrate (5 µg·kg^-1^·h^-1^), and muscular relaxation was maintained with pancuronium bromide (0.2 mg·kg^-1^·h^-1^). The adequate depth of anesthesia during surgery was evaluated with maintenance of physiological variables (heart rate and arterial pressure) and absence of reflexes (corneal and hind limb flexion response), as well as unresponsiveness to stimuli during manipulation. Supplementary boluses of 5 µg/kg fentanyl and 0.125 mg/kg midazolam were administered as necessary.

Immediately before handling and thereafter every 6 h, all the animals received one gram of cephalothin IV as antimicrobial prophylaxis. The right external jugular vein was dissected for introduction of a pulmonary artery catheter, which was guided to the pulmonary artery by visualization of the pressure curves. A double-lumen catheter (12F) was inserted in the left jugular vein for fluid and drug administration and to collect blood during the hemorrhage period. A 5F catheter (PV2015L20, Pulsion Medical Systems, Germany) was inserted into the right femoral artery for thermodilution measurements and pulse contour analysis and connected to the Infinity^®^ PiCCO SmartPod™ XL monitor (Infinity Delta XL, Dräger).

Through a midline laparotomy, a cistostomy was performed, and a vesical catheter inserted. A Laser DopplerFlowmetry catheter (Transonic Systems Inc., USA) was inserted in the terminal ileum to measure intestinal microcirculatory perfusion and the laparotomy was closed. After instrumentation, the animals were allowed to stabilize without interventions for 1 h and a continuous infusion of 5 mL·kg^-1^·h^-1^ of lactated Ringer's solution was maintained throughout the experiment.

Systemic pressures were measured with quartz transducers (Edwards Critical Care, USA) and displayed continuously on a multi-modular monitor (Infinity Delta XL, Dräger), which also measured heart rate, oxygen saturation and end-tidal CO_2_. Continuous central venous oximetry was measured by spectrophotometry and continuous cardiac output was measured by thermodilution (Vigilance™, Edwards, USA).

### Blood collection and processing

Immediately after the stabilization period, the animals were submitted to a controlled hemorrhage of 25% of the blood volume [estimated as body weight (kg) × 70 mL) during 40 min (23). The blood was collected into citrate-phosphate-dextrose-adenine 1 (CPDA-1) blood bags (Fresenius Hemo Care, Brazil). The whole blood was centrifuged at 1130 *g* for 16 min at 4°C (Beckman-Spinchron 15 centrifuge, Beckman Coulter, USA) and separated into plasma and red blood cell using a manual Fenwal plasma extractor (Baxter, USA). In total, two RBC and two plasma units were obtained from each animal. The plasma units were discarded and the RBC units stored at 4°–8°C for 14 days. The target temperature was tracked by a temperature recorder (iButton DS1921g, USA).

After 30 min of hemorrhage and before fluid infusion, the animals were allocated in two groups: a) Control group (n=8) - fluid replacement using lactated Ringer's solution equivalent to 3 times the removed volume in up to 2 h; b) Transfusion group (n=8) - infusion of two packed RBC units (collected from another animal 14 days before) in a period of 2 h and lactated Ringer's solution in a volume correspondent to 3 times the difference between removed volume and transfused volume. RBC units were cross-matched using a visual agglutination test and were obtained from a single porcine donor. After fluid resuscitation, the animals were observed for 6 h and, at the end of the experiments, were sacrificed with potassium chloride overdose after deepening of anesthesia.

We demonstrated in a previous study that swine RBC stored under human standard conditions for 14 days had conserved viability as evaluated *in vitro* using free hemoglobin and hemolysis index and *in vivo* using RBC labeled with sodium chromate (24). The 14-day storage time was chosen based on previous studies, which demonstrated that the potential half-life of transfused swine RBC labeled with radioactive sodium chromate ranges from 14 to 20 days (25).

### Hemodynamic and respiratory variables acquisition

The hemodynamic parameters measured during every condition were mean arterial blood pressure, central venous pressure, pulmonary artery pressure, heart rate, continuous central venous oximetry, cardiac output, stroke volume variation, global ventricle ejection fraction, global end diastolic volume and extravascular lung water index. Respiratory variables acquired were expired minute volume, tidal volume, respiratory rate, peak and plateau (calculated as the mean of 5 consecutive respiratory cycle plateau pressure measurements with a static inspiratory pause of 2 s) pressures, PEEP, end-tidal carbon dioxide concentration, inspiratory oxygen fraction and arterial oxygen saturation.

Pressure × volume (PV) curves of the respiratory system were done at baseline, immediately after resuscitation and after 3 and 6 h of fluid replacement, through a low flow method available in the mechanical ventilator, as described (26). The inspiratory and expiratory work estimated as the areas under the inspiratory and expiratory limbs of the PV curve were also analyzed. The difference between expiratory and inspiratory work was considered as the energy spent with lung hysteresis. All areas were normalized to the area of the smallest rectangle capable of enclosing the whole PV curve (27,28). The area of this rectangle was considered as the potential energy of the pulmonary envelope, and normalization was necessary due to the heterogeneity of viscoelastic properties of the respiratory system of the animals (29).

### Laboratory analysis

Arterial samples were collected before and after the conditions described above for measurement of blood gases and arterial lactate on a blood gas analyzer (ABL 700 Radiometer, Denmark). Venous oximetry was calibrated *in vivo* with gas analysis of a mixed venous sample.

### Inflammatory response

Before the induction of hypovolemia, and after 3 and 6 h of resuscitation, blood samples were withdrawn from arterial catheters for measurement of interleukin (IL)-6, IL-10, IL-1, and IL-21 concentrations, using porcine-specific ELISA kits for IL-6, IL10, IL-1 and a non-specific kit for IL-21, according to the manufacturer's instructions (R&D Systems, USA). We also evaluated plasma nitrate concentrations by chemiluminescence technique (Sievers NO analyzer model 280; Sievers Instruments Inc., USA) at the same time-points. The lung was collected and frozen immediately after sacrifice for the preparation of tissue homogenates (26) and pulmonary concentrations of the same cytokines and nitrate were measured using these techniques. The results were normalized for pulmonary protein concentration.

The evaluation of gene expression for several inflammatory mediators in the pulmonary tissue was carried out through reverse transcription quantitative polymerase chain reaction (RT-qPCR). For these experiments, fragments of frozen pulmonary tissue were homogenized; total RNA was isolated using the “RNAeasy Minikit” (QIAGEN Sciences, USA) following manufacturer's instructions. After cDNA synthesis from 1 μg of RNA with the “SuperScript III First-Strand Synthesis Systems” (Life Technologies, USA) the cDNA samples were submitted to qPCR in the SDS7900HT (Life Technologies). GAPDH and HPRT1 were used as control genes. The probes and primers for each of the genes evaluated are as follows: IL-1β, sense: GGTTTCTGAAGCAGCCATGG, antisense: GATTTGCAGCTGGATGCTCC, Probe 5′(FAM)-AAAGAGATGAAGTGCTGCACCCAAAACCTG-(TAMRA)3′; IL-10, sense: TTGGAGCTTGCTAAAGGCACT, antisense: CGGCGCTGTCATCAATTTCT, Probe 5′(FAM)-CACCTCCTCCACGGCCTTGCTCTT-(TAMRA)3′; IL-21, Ss03384710_u1; iNOS Ss03374608_u1; TNF alpha Ss03391318_g1; HPRT-1 Ss03388274_m1; GAPDH Ss03375629_u1 (Life Technologies).

### Statistical analysis

Respiratory system compliance was our primary end-point. Unlike other cardiovascular and inflammatory parameters, previous clinical (17) and experimental (15) studies have demonstrated alterations on this variable after stored RBC transfusion. Based on our previous experimental work that measured this variable, we estimated that a study with 16 animals (8 animals per group) would have an 80% power to detect a difference of 25% in respiratory system compliance between groups, considering a normal value of 35 mL/mmHg and a within-group standard deviation of 5 mL/mmHg (29).

Data were tested for normality using Shapiro-Wilk test and are reported as means±SD or median (p25-p75) according to their distribution. Evolutive data (hemodynamic, oxygenation, respiratory) are reported as mean and SD over time and were compared using two-way analysis of variance (ANOVA). Bonferroni correction for multiple comparisons was used, and for five-time point comparisons (baseline, hemorrhage, immediate resuscitation, 3 and 6 h), significance was considered when P≤0.01 for the three levels of the analysis (between, within, and factor × time interaction). *Post-hoc* analyses were performed with the Tukey test. Non-parametric data were evaluated with the Friedman test and Tukey *post-hoc* analysis. The commercially available SigmaStat 2.0 statistical package (Systat Software, USA) was used.

## Results

**Figure 1.**
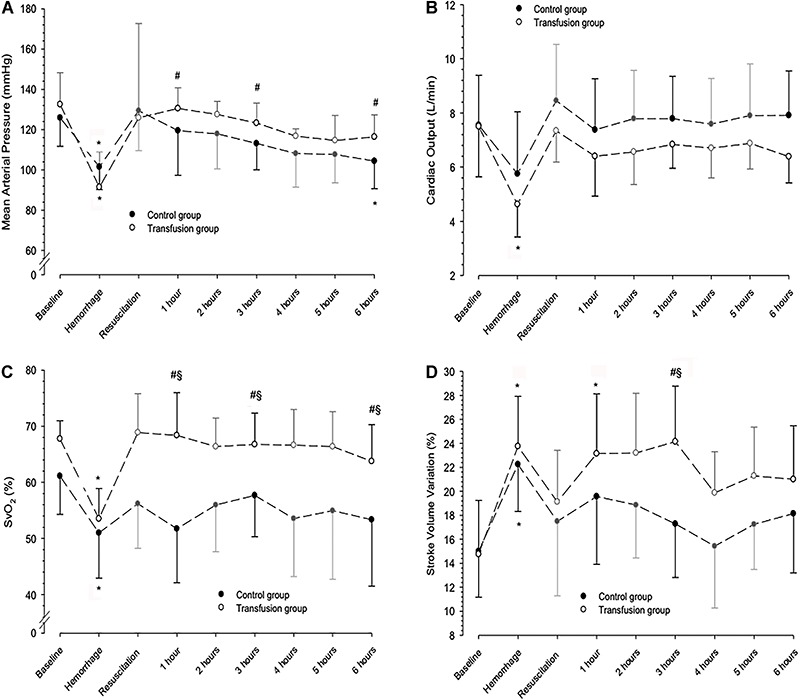
Sequential hemodynamic parameters of the groups during the study. *Panel A*, Mean systemic arterial blood pressure (ANOVA between factor P=0.156, within factor P<0.001 and factor-time interaction P=0.054). *Panel B*, Cardiac output (ANOVA between factor P=0.013, within factor P<0.001 and factor-time interaction P=0.773). *Panel C*, Mixed venous oxygen saturation (SvO_2_) (ANOVA between factor P<0.001, within factor P<0.001 and factor-time interaction P=0.019). *Panel D*, Stroke volume variation (ANOVA between factor P=0.037, within factor P<0.001 and factor-time interaction P=0.207). Gray lines and markers denote all data recorded for 8 animals per group. Black markers denote the data that were chosen *a priori* to be analyzed. *P<0.05 *vs* baseline, ^#^P<0.05 *vs* control group, ^§^P<0.05 *vs* hemorrhage (*post-hoc* Bonferroni correction).

**Figure 2.**
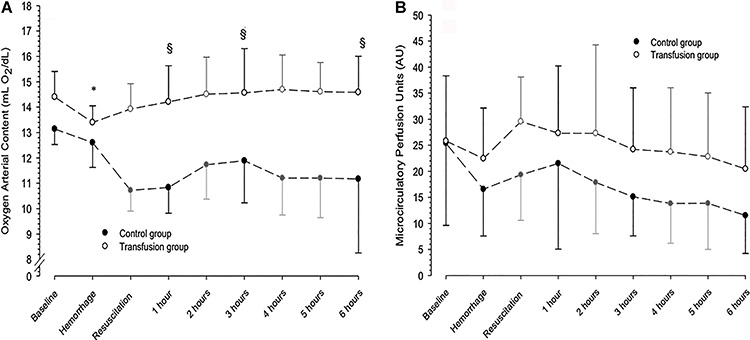
Sequential oxygenation and perfusion parameters of the groups during the study. *Panel A*, Arterial oxygen content (ANOVA between factor P<0.001, within factor P=0.039 and factor-time interaction P=0.015). *Panel B*, Arbitrary microcirculatory perfusion units (ANOVA between factor P=0.026, within factor P=0.146 and factor-time interaction P=0.841). Gray lines and markers denote all data recorded for 8 animals per group. Black markers denote the data that were chosen *a priori* to be analyzed. *P<0.05 *vs* baseline, ^§^P<0.05 *vs* hemorrhage (*post-hoc* Bonferroni correction).

As expected, hypovolemia was associated to significant reductions in mean arterial pressure, cardiac output, mixed venous oxygen saturation (Figure 1A, B and C) in both groups. In addition, hypovolemia significantly increased stroke volume variation (Figure 1D). In both groups, fluids and RBC transfusion partially or fully reestablished these parameters. However, the transfused group had higher values of central venous oxygen saturation (Figure 1C) and arterial oxygen content (Figure 2A). There was a significant increase in hemoglobin and hematocrit level (Table 1) of the transfused group compared to the control animals. Transfusion did not influence the microcirculation and perfusion as assessed by Laser Doppler flowmetry (Figure 2B) and lactate concentrations (Table 1).

**Table 1.** Hemodynamic and laboratory variables of the groups during the study period.

Variable/Group	Baseline	Hemorrhage	Resuscitation			P value
			1 h	3 h	6 h	
**Cardiac output**						
Heart rate (bpm)						
Transfusion	111±16	118 ±17	114±11	119±12	124±13	0.10¶
Control	108±26	133±32	130±29	140±31	135±23	0.019^&^
Cardiac Index (mL/kg)						
Transfusion	106±27	65±17*	90±22	97±13	91±15§	<0.001¶
Control	113±28	86±32	111±27	117±24	119±25	<0.001^+^
GEDVI						
Transfusion	557±99	449±87	512±60	504±79	462±109	0.054¶
Control	630±139	507±148	552±130	529±118	528±130	0.043^&^
**Left ventricle**						
PAOP (mmHg)						
Transfusion	8±3	7±4	8±3	8±3	7±4	0.475¶
Control	6±2	5±1	8±2	6±2	7±4	0.110^&^
SVRI [dynes·s^-1^·(cm^5^) ^-1^·kg^-1^]						
Transfusion	100±32	110±31	115±29§	98±16	99±21§	0.109¶
Control	89±24	101±33	87±25	76±18	69±17	<0.001^&^
LVSWI [(mL·mmHg)·kg^-1^·beat^-1^)]						
Transfusion	1.5±0.2	0.6±0.2*	1.3±0.2^#^	1.2±0.1^#^	1.1±0.2^*#^	<0.001¶
Control	1.7±0.3	0.8±0.2*	1.3±0.4*^#^	1.2±0.2*	1.1±0.2*	0.153^&^
**Right ventricle**						
PAPm						
Transfusion	25±3	17±6*	24±4	26±7^#^	27±6^#^	0.003¶
Control	25±5	19±4	23±3	26±8	22±6	0.538^&^
CVP (mmHg)						
Transfusion	6±1	5±1	6±1	6±1	6±1	0.082¶
Control	5±2	3±2§	4±2	4±2§	5±2	<0.001^&^
PVRI [dynes·s^-1^·(cm^5^) ^-1^·kg^-1^]						
Transfusion	13±3	12±6	14±3	15±7	17±2	0.851¶
Control	13±5	13±6	11±3	14±8	10±5	0.111^&^
RVSWI [(mL·mmHg)·kg^-1^.beat^-1^)]						
Transfusion	0.24±0.08	0.09±0.06*	0.20±0.07^#^	0.23±0.09^#^	0.21±0.09^#^	<0.001¶
Control	0.29±0.05	0.14±0.05*	0.23±0.06	0.24±0.03^#^	0.20±0.04	0.084^&^
**Perfusion and laboratory**						
Urinary flow (mL/kg/h)						
Transfusion	1.6±0.9§	0.7±0.5	2.3±0.9	2.4±1.6	1.8±0.8	0.006¶
Control	3.3±1.7	1.4±0.9*	3.0±2.1	2.6±1.7	1.9±0.9	0.027^&^
Cumulative fluid balance (mL/kg)						
Transfusion	20±6	-	28±7§	37±6*§	46±6*§+	<0.001¶
Control	20±3	-	43±4*	50±8*	59±11*^++^	<0.001^&^
Lactate (mEq/L)						
Transfusion	2.0±0.6	2.1±0.6	1.7±0.5	1.3±0.4	1.2±0.2	<0.001¶
Control	2.1±0.9	2.6±0.6	1.7±0.6	1.3±0.4^#^	1.3±0.5^#^	0.467^&^
SBE (mEq/L)						
Transfusion	4.0±3.6	1.5±4.3§	3.8±2.6	3.9±3.2	4.3±2.7	0.182¶
Control	5.7±1.8	4.4±2.2	5.0±1.7	5.6±2.0	6.3±2.1	0.004^&^
Hemoglobin						
Transfusion	10.7±0.7	9.9±0.4	10.7±0.9§	11.2±1.1^#^§	11.4±0.9§	0.029¶
Control	10.0±0.7	9.2±1.1	8.1±0.7*	9.0±1.1	8.9±1.2	<0.001^&^
Hematocrit						
Transfusion	33±2§	31±1	3±2§	3±3§	35±2§	0.459¶
Control	27±9	29±3	25±2	28±3	28±4	<0.001^&^

Data are reported as means±SD for 8 animals per group. GEDVI: global end diastolic volume indexed; PAOP: pulmonary artery occlusion pressure; SVRI: systemic vascular resistance index; L*VS*WI: left ventricle stroke work index; PAPm: pulmonary arterial pressure media; CVP: central venous pressure; PVRI: pulmonary vascular resistance index; R*VS*WI: right ventricle stroke work index; SBE: standard base excess. ^¶^ANOVA two-way within factor. **^&^**ANOVA two-way between factor. There was no factor *vs* time interaction. *P<0.05 *vs* baseline (Tukey's *post-hoc* analysis); ^#^P<0.05 *vs* hemorrhage (Tukey's *post-hoc* analysis); ^§^P<0.05 *vs* control group (Tukey's *post-hoc* analysis); ^+^P<0.05 *vs* 1 h resuscitation (Tukey's *post-hoc* analysis).

Table 2 demonstrates the effects of RBC transfusion on respiratory function. We were unable to identify any acute effect of transfusion in the several variables evaluated. Importantly, transfusion did not induce a significant effect on extravascular thermal volume index, which is a surrogate for extravascular lung water (30). There was a significant increase in the energy spent on inspiratory work and pulmonary hysteresis for the control group (Table 2). This may be representative of increased cumulative fluid balance in the control group (Table 1). Transfusion *per se* did not cause significant alterations in these variables.

**Table 2.** Respiratory parameters of the groups during the study period.

Variable	Baseline	Hemorrhage	Resuscitation			P value
			1 h	3 h	6 h	
P/F ratio						
Transfusion	381±43	372±31	316±49	288±69	255±85*	<0.001¶
Control	343±70	332±79	293±77	268±65	247±73*	0.055^&^
EtCO2 (mmHg)						
Transfusion	34±4^§^	34±5	35±3	38±2	37±3	0.346¶
Control	37±3	37±3	37±5	39±2	37±6	0.022^&^
Ppeak (cmH2O)						
Transfusion	21±1	20±2	22±4	24±7	25±8	0.105¶
Control	24±4	23±4	24±4	26±6	26±5	0.061^&^
Pplateau (cmH2O)						
Transfusion	17±1	16±2	17±3	19±5	20±6	0.237¶
Control	17±4	17±4	18±2	19±3	20±4	0.739^&^
Pmean (cmH2O)						
Transfusion	10±0.4	9±0.4	10±1	10±2	11±3	0.448¶
Control	11±1	11±2	10±1	11±1	12±1	0.042^&^
Cstat (mL/mmHg)						
Transfusion	44±5	49±8	46±10	43±11	41±12	0.040¶
Control	46±15	43±9	41±8	38±9	37±9	0.110^&^
Airway resistance (mmHg·L^-1^·s ^-1^)						
Transfusion	7±2	6±1	8±2	8±2	8±2	0.090¶
Control	7±1	7±2	8±2	9±3	9±3	0.287^&^
Inspiratory energy (%)						
Transfusion	56±2	-	56±2	54±2	53±1§	0.479¶
Control	57±3	-	57±2	57±6	57±5	0.009^&^
Expiratory energy (%)						
Transfusion	43±4	-	43±3	41±2	40±3§	0.023¶
Control	40±7	-	42±6	36±4	35±4^#^	0.008^&^
Hysteresis energy (%)						
Transfusion	13±4	-	13±3	13±2§	13±3§	0.257¶
Control	17±7	-	15±5	21±7	21±5	<0.001^&^
ETVI (mL/kg)						
Transfusion	6.2±1	5.4±1	6.4±1.2	6.8±1.6	6.5±0.9	0.557¶
Control	8±2	7.2±2.1	7.8±2.8	8±2.6	7.8±2.5	<0.001^&^

Data are reported as means±SD for 8 animals per group. Inspiratory energy, expiratory energy and hysteresis energy were calculated using the pressure × volume (P×V) curve of the respiratory system normalized to the area of the rectangle enclosing the P×V curve 29. P/F ratio: PaO_2_/FiO_2_ ratio; EtCO_2_: end-tidal carbon dioxide concentration; Ppeak: peak airway pressure; Pplateau: plateau airway pressure; Pmean: mean airway pressure; Cstat: respiratory system static compliance; ETVI: extra-vascular thermal volume index. There is no factor *vs* time interaction. ^¶^ANOVA two-way within factor. ^&^ANOVA two-way between factor. *P<0.05 *vs* baseline, Tukey's *post-hoc* analysis. ^§^P<0.05 *vs* Control group, Tukey's *post-hoc* analysis, ^#^P<0.05 *vs* 1 h resuscitation, Tukey's *post-hoc* analysis.

**Figure 3.**
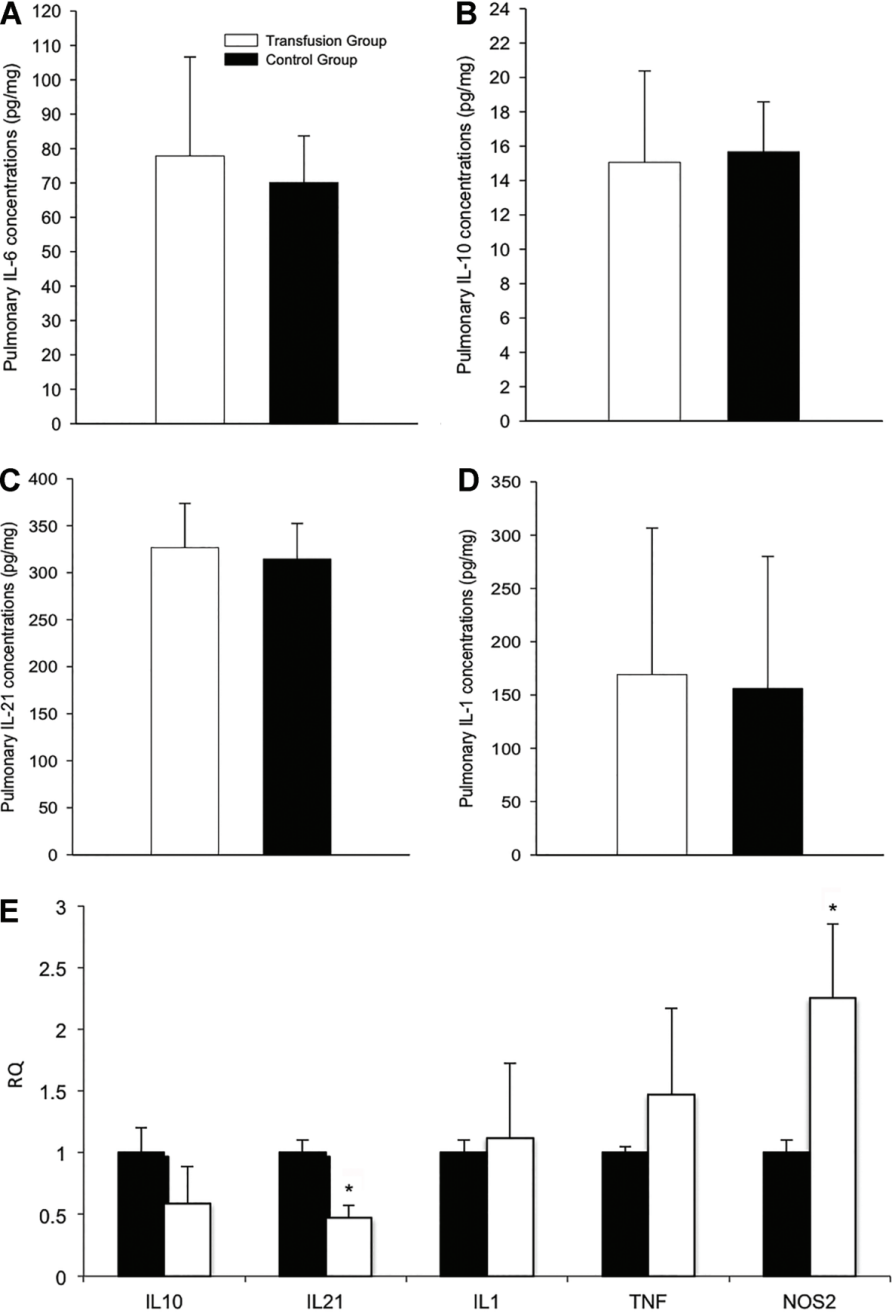
Inflammatory parameters of the groups during the study. *Panel A*, pulmonary IL-6 concentrations (P=0.401, *t*-test). *Panel B*, pulmonary IL-10 concentrations (P=0.817, *t*-test). *Panel C*, pulmonary IL-21 concentrations (P=0.567, *t*-test). *Panel D*, pulmonary IL-1 concentrations (P=0.844, *t*-test). *Panel E*, Relative mRNA quantification (RQ) for cytokines and NOS2 in lung tissue. Data are reported as means±SD for 8 animals per group. Animals were sacrificed 6 h after the end of transfusion. *P<0.05 *vs* Control (*t*-test).

**Figure 4.**
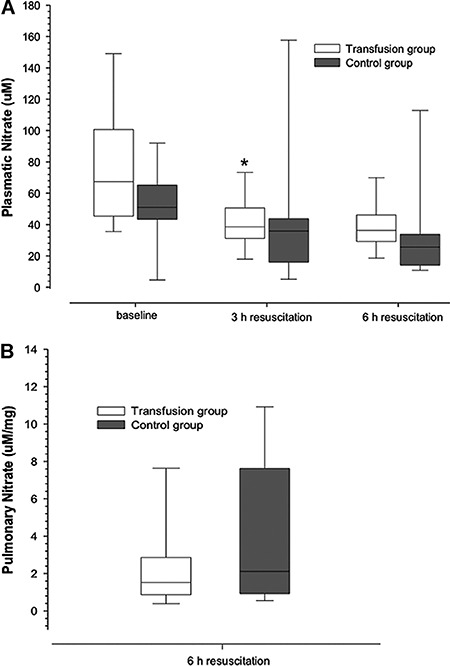
Nitrate concentrations of the groups during the study. *Panel A*, plasma nitrate concentrations of both groups (P=0.021, Friedman test). *P<0.05 *vs* baseline (Tukey *post-hoc* analysis). *Panel B*, pulmonary nitrate concentrations of both groups (P=0.505, Mann-Whitney rank sum test). Data are reported as medians and interquartile range for 8 animals per group. Animals were sacrificed 6 h after the end of transfusion.

The plasma concentrations of the several evaluated cytokines before and after fluid replacement and RBC transfusion were below the ELISA detection threshold for the vast majority of animals studied (data not shown). In Figure 3A-D, cytokine concentrations in the lung tissue are reported. There was no difference between transfused and non-transfused groups. A small although statistically significant difference between transfused and control groups was obtained for relative mRNA quantitation of NOS2 and IL-21, with a two-fold increase in the former and a 50% decrease in the latter in the transfused animals compared to controls (Figure 3E). Plasma and pulmonary concentrations of nitrate are reported in Figure 4 and no significant differences were observed between groups.

## Discussion

In this study, we describe a swine experimental model of stored homologous RBC transfusion that may be used to study the clinical and mechanistic effects of this strategy commonly used in clinical practice. In addition, we demonstrate that stored RBC transfusion is not related to acute clinically significant effects on pulmonary function, microcirculation and lung inflammation in otherwise normal animals.

The association between pulmonary complications and RBC transfusion is described mainly in observational studies. An early trial done in anemic preterm infants demonstrated that transfusion induced a reduction in respiratory compliance, increments in pulmonary vascular resistance and increased work of breathing. However, these alterations may not be the result of transfusion *per se* but may have occurred due to either volume overload or increases in lung water content (17). These deleterious effects were not reproduced in other studies, since transfusion decreased the work of breathing without any harmful effect on gas exchange in both patients with chronic obstructive pulmonary disease and in anemic controls (31). A study in patients undergoing cardiopulmonary bypass did not identify significant alterations in gas exchange in those with multiple transfusions, compared to few transfusions or non-transfused patients (32). Another study in cardiothoracic surgery patients showed a dose-dependent increase in pulmonary capillary permeability (33). Additionally, a study in healthy volunteers demonstrated subclinical acute pulmonary alterations one hour after autologous RBC transfusion evidenced by impaired oxygen gas exchange, however without any significant effect in arterial oxygen pressure (34). In the present report, we did not find any clinically significant effect of RBC administration on gas exchange and respiratory mechanics thus suggesting that this strategy of transfusion does not induce acute respiratory derangement in normal lungs.

Few studies in the literature assessed the effects of RBC transfusion on inflammatory response, with discordant results. In normal rats, blood transfusion did not induce lung inflammation, which is similar to our results (35). However, pre-treatment with endotoxin significantly amplified the inflammatory effects of transfusion with increase in neutrophil counts and chemokine concentrations in the airways (34). A study that developed a swine model similar to ours demonstrated that transfusion of old stored RBC (mean 37 days of storage) is associated with significant decrements on renal function and augmentation of inflammatory response in a swine cardiopulmonary bypass model (15). However, these authors used a very long storage time since the half-life of homologous transfused RBC in swine is up to 20 days (25). Moreover, the RBC average life spans are shorter in pigs than in humans (86 *vs* 120 days) (36), which suggests that the effects observed in this study would be very difficult to reproduce in human conditions. In our study, the only significant effects of RBC transfusion on pulmonary inflammation were the modifications on pulmonary gene expression of NOS2 and IL-21. NOS2 has been consistently correlated to inflammation and IL-21 is a recently studied cytokine that interacts with IL-10 in the regulation of anti-inflammatory response (37). However, these alterations were not followed by significant changes in the downstream pathways of these mediators, since we could not identify variations in the IL-21 protein or nitrate concentration in pulmonary tissue. It is possible that the effect of RBC on lung inflammation would augment with the exposure to a concomitant insult like sepsis or lung injury or by a more extended period of observation.

Regarding clinical situations, a previous study detected increases in plasma concentrations of IL-6 and IL-8 after RBC transfusion in critically ill patients (38). In addition, a study more specifically focused on the pulmonary effects of RBC administration suggested that in patients concomitantly submitted to cardiopulmonary bypass and blood transfusion, multiple transfusions were associated with increased concentrations of cytokines and coagulation factors in the bronchoalveolar lavage (32). However, a more recent study evaluating lung function in healthy volunteers after fresh and stored autologous RBC transfusion could not find any significant changes in hemodynamic, respiratory and inflammatory variables. These results demonstrate the controversy of the theme (39).

An important discussion in the field of transfusion medicine is whether the storage lesion is associated with clinical unfavorable outcomes (40). Observational studies and experimental data have demonstrated an association between storage injury, inflammatory response and grim prognosis (4). However, clinical trials and a subsequent meta-analysis demonstrated no cause-effect relationship between transfusion of old blood and significant adverse effects or mortality, compared to flesh blood (40). Thus, this question is still unsolved. The model presented here can help to clarify the mechanisms related to storage lesion in future experiments with different blood preservation periods.

Our study has some major strengths, such as the extensive evaluation of hemodynamics, pulmonary function and the assessment of possible mechanistic pathways associated with transfusion-related lung effects. On the other hand, a limitation of our data is the possibility of type II errors in the analysis, due to a relatively small sample. Another limitation is the relatively short period of observation after blood transfusion. Deleterious effects of RBC transfusion might occur later, although previous experimental studies demonstrated that harmful effects of transfusion occurred as early as 1.5 and 4 h after RBC administration (21). Besides, we did not evaluate the effect of RBC transfusion in animals previously submitted to other injuries like sepsis, lung dysfunction or anemia or using different blood storage periods, so the clinical translation of our results is not straightforward. In previously “activated” lungs, the effects of blood transfusion might be potentiated, as discussed earlier. However, our major objective here was to evaluate the independent effects of blood transfusion in otherwise healthy lungs and to provide a feasible and consistent model of RBC transfusion that may be used in experimental studies in intensive care scenarios.

We successfully developed an experimental model of RBC transfusion in pigs that may be used to carry out mechanistic research aiming at cardiovascular and respiratory functions. Importantly, we also demonstrated that transfusion of RBC stored for 14 days was not associated with acute clinically significant cardiac, respiratory and pulmonary inflammatory effects in otherwise healthy pigs submitted to hypovolemia. Subsequent studies are required to assess the pathophysiological consequences of adding RBC transfusion to experimental models of critical illnesses.
